# Contribution of G.A. Ilizarov to bone reconstruction: historical achievements and state of the art

**DOI:** 10.1007/s11751-016-0261-7

**Published:** 2016-07-18

**Authors:** Alexander V. Gubin, Dmitry Y. Borzunov, Larisa O. Marchenkova, Tatiana A. Malkova, Irina L. Smirnova

**Affiliations:** Russian Ilizarov Scientific Center for Restorative Traumatology and Orthopaedics, 6, M. Ulianova Street, Kurgan, Russian Federation 640014

**Keywords:** Ilizarov method, Bone regeneration, Distraction, Compression, External fixation

## Abstract

Methodological solutions of Prof. G.A. Ilizarov are the core stone of the contemporary bone lengthening and reconstruction surgery. They have been acknowledged in the orthopaedic world as one of the greatest contributions to treating bone pathologies. The Ilizarov method of transosseous compression–distraction osteosynthesis has been widely used for managing bone non-union and defects, bone infection, congenital and posttraumatic limb length discrepancies, hand and foot disorders. The optimal conditions for implementing distraction and compression osteogenesis were proven by numerous experimental studies that Prof. G.A. Ilizarov organized and supervised at a large orthopaedic research institute in Kurgan. The tension stress effect on regeneration and growth of tissues was thoroughly investigated with radiographic, histological and biochemical methods. The impact of the Ilizarov method on the progress of bone lengthening and reconstruction surgery could be called revolutionary.

## Introduction

**Fig. 1:**
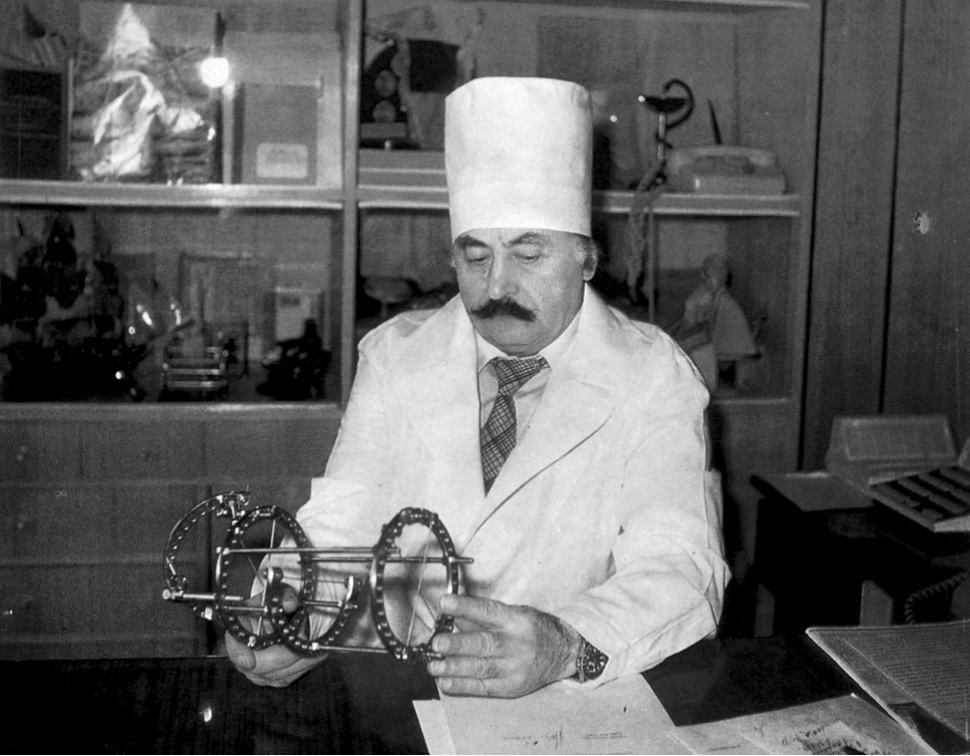
Prof. G.A. Ilizarov (1921–1992) in his study. Courtesy of the Centre’s museum

Almost 65 years have passed since Prof. G.A. Ilizarov (Fig. 1) introduced his apparatus for external bone fixation and began to develop the techniques for managing bone injuries and orthopaedic diseases [1–7]. Nowadays, his methodological solutions are the core stone of limb lengthening and reconstruction surgery and have been acknowledged in the orthopaedic world as one of the greatest contributions to treating bone pathologies [5–7].

He started to develop his ideas of external fixation in the middle of the last century when he was a rural surgeon in the Kurgan region of Russia. In the 1970–1980s, his ideas grew into a profound fundamental research and clinical work conducted at one of the biggest orthopaedic centres of the world that specializes in bone reconstruction and is his brainchild.

The first Ilizarov external fixator was used for bone fragment fixation to external rings through the wires that transfixed the bone and were able to produce longitudinal compression or distraction in a fractured or osteotomized bone with external threaded rods [7]. The reduction wires (olive wires) and units (hinges) were designed later [1] and provided control of bone fragment positions. Thus, the wires that crossed inside the bone at angles could be guided with the adjustments of the external elements in order to correct bone angulation, translation or torsion. And that was the zest that has resulted in numerous solutions for bone reconstruction with the external apparatus.

## Scientific study of distraction osteogenesis

Prof. G.A. Ilizarov first reported on a positive impact of bone distraction on osteogenesis at the All-Russia Congress of Orthopaedic and Trauma Surgeons in 1963 [8]. Later, bone regeneration in the process of distraction osteogenesis was intensively studied under his guidance by the researchers at a special experimental department of the scientific institute (former name KNIIEKOT); he had founded in 1971. Those experiments found optimal conditions for implementing distraction osteogenesis that include stable fixation of bone fragments with the external apparatus, a non-invasive corticotomy, a daily distraction rate of 0.75–1 mm/day in three or four increments [1–7], limb weight bearing and joint motion that are also an obligatory condition in his treatment system.

**Fig. 2:**
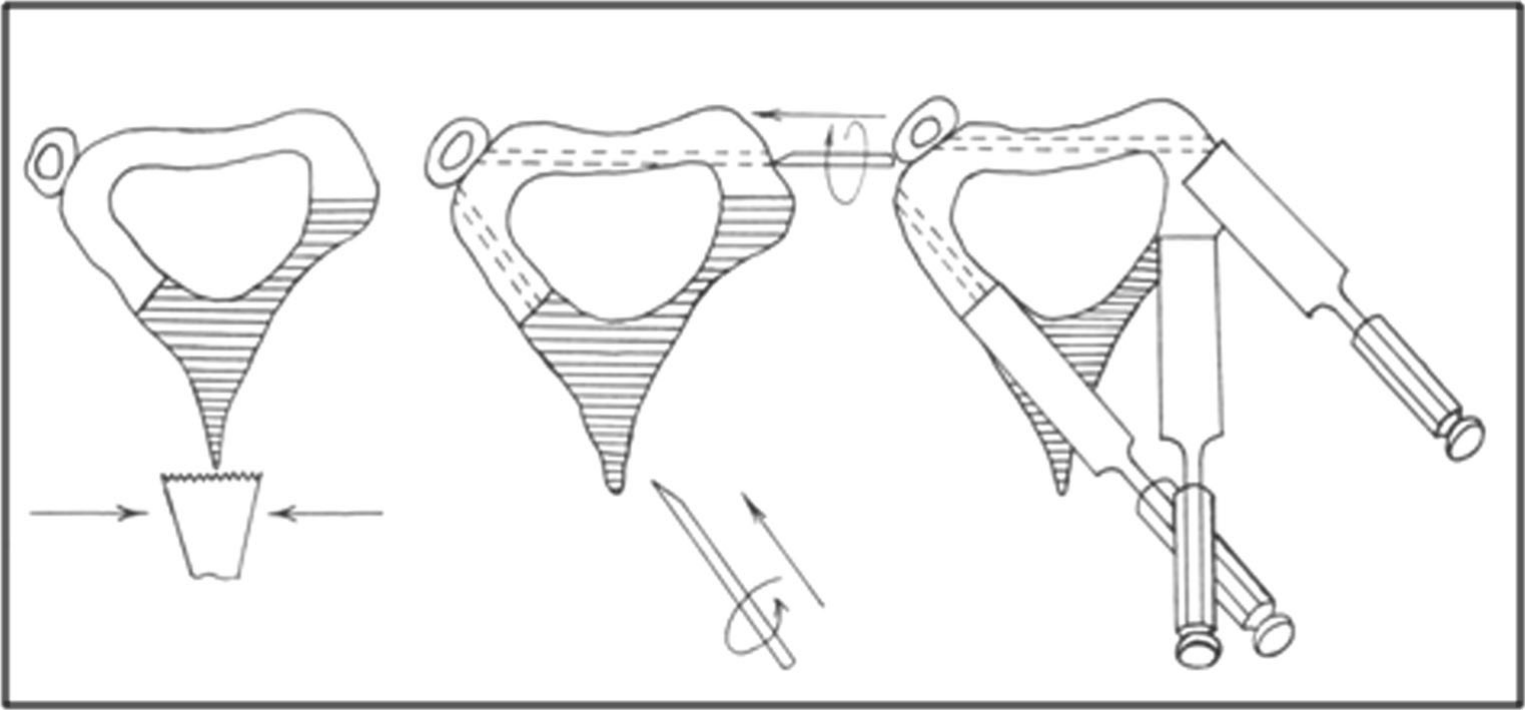
A variant of tibial corticotomy which is performed from a 1.5-cm incision on the anteromedial surface. Drilling of the posterolateral cortex with a Kirshner wire is produced tangently to provide a corticotomy direction line

The tension stress effect on regeneration and growth of tissues (USSR discovery certificate dated 23.04.1989) that is induced with the forces of the external apparatus was thoroughly investigated with radiographic, histological and biochemical methods [1–6]. Canine experimental models were used to reveal the potential of guided bone distraction on bone tissue growth and the dependence of its quality and quantity on blood supply, rates and rhythm of distraction, the impact of injury to the osteogenic elements of a tubular bone such as bone marrow, endosteum and periosteum, nutrient artery and on bone fragment fixation rigidity. It was proven that the best surgical methods to break the bone and preserve the medullary canal content were corticotomy and closed flexion osteoclasis instead of osteotomies that injure the content [1]. Corticotomy has become a classical way of breaking a bone for lengthening or deformity correction with the Ilizarov apparatus that is produced from a small incision using a chisel to transect the cortex two-thirds around the bone and accomplish osteolasis by turning the chisel within the cortex or by counter rotating the rings (Fig. 2).

It was also discovered that along with bone tissue growth other biological tissues of the limb (muscles, nerves, ligaments, tendons and skin) responded to gradual distraction. Gradual distraction induced or supported stimulation of their growth, biosynthetic activity and energy interchange.

It was revealed that the formation of a longitudinal distraction regenerate was accompanied by generation of a large number of vessels of various sizes in the bone itself and in the surrounding tissues. At the end of 1970s, this effect started to be used for stimulation of regional vascularity in ischemic limb diseases by formation of a longitudinal bone split for its transverse traction [9]. Transverse bone distraction was the solution for bone thickening [10] and fibular tibilization in subtotal tibial defects and extensive tibial defects with atrophic tibial fragments when traditional bone plasty is impossible or difficult to realize [11].

Ilizarov transferred his principles to cancellous bone distraction and experimented on dogs to lengthen vertebrae and manage cranial defects. Under his guidance, the techniques for cranial defect (author’s certificate from 23.10.83) and spine disorders management with a special apparatus for external fixation of the spine (patented on 06.02.85) started to be developed and later were used at the Centre. Unfortunately, the findings of those experimental studies conducted by him and Centre’s researchers were not published in the international journals, but his successors issued a book on craniofacial distraction [12].

## Transosseous osteosynthesis

Ilizarov named his method *transosseous compression distraction osteosynthesis* and formulated its principles [1, 2]. Worldwide, it is simply called the Ilizarov method though the method is a collective result of a large team of talented scientists, surgeons and engineers, he had gathered around him. It is a system of techniques that induce *compression* or *distraction* (or the combination of both forces) by moving bone fragments via *transosseous* wires with the adjustments of the external ring fixator for bone union, growth or spatial transformation that finally ends in *osteosynthesis,* consolidation and new bone remodelling. These techniques are named according to the forces applied. The summary of their use for skeletal injuries, their complications, congenital disorders, degenerative diseases and tumours is given in Table 1.

**Table 1:** Compression and distraction techniques

Compression Longitudinal, side-to-side (or combined) supportive* unifocal, bifocal (multifocal)	Distraction Longitudinal, transverse Unifocal, bifocal (multifocal)
Fractures Non-union (congruent bone ends) Arthrodesis	Bone lengthening (LLD, achondroplasia) Bone thickening Deformity correction stiff non-union

Combined compression–distraction or distraction–compression			
Unifocal		Bifocal (multifocal)	
Simultaneous	Sequential	Simultaneous	Sequential
Stiff hypertrophic non-union with angulation (LLD** up to 1 cm)	Stiff hypertrophic non-union + LLD knee lengthening arthrodesis	Non-union and defects + LLD	Non-union defects (bone transport)

* Supportive compression is used every 7–10 days in managing non-union and bone defect

** *LLD* limb length discrepancy

In the Soviet Union, there was (and continues to exist in Russia) a system for certification of inventions and methods by the governmental bodies (former *USSR State Committee on Inventions and Discoveries*, nowadays *Rospatent*). The devices invented by Ilizarov were patented, while the techniques developed were certified and published in the *USSR Bulletin of Inventions* (Table 2). Furthermore, Ilizarov submitted papers on his experimental and clinical studies to the most prominent journals of the Soviet Union such as *Ortopedia, Travmatologia y Protezirovanie* and *Vestnik Khirurgii im. Grekova* indexed by the US National Library of Medicine. Therefore, the priority of his techniques can be traced back historically [10, 13–24].

**Table 2:** Certification of the Ilizarov techniques by the USSR certification board and publications in the indexed journals

	Certification of the method	Publication of clinical studies
Long bone fracture union and pseudarthrosis (including complicated by infection)	Applied on 09.06.52 Published on 17.08.1954	November 1972 [13]
Long bone defects (including complicated by osteomyelitis) (bone transport)	07.01.1967 Published on 07.09.1971	September 1969 [14, 15] November 1973
Hip arthrodesis, femur lengthening	Applied on 07.01.67 05.10.1971	June 1969, May 1973 [16, 17]
Congenital pseudoarthrosis of the tibia	n/a	February, 1969 [18]
Long bone lengthening	Applied on 21.03.1971 Published on 05.10.1974	March, 1969 [19]
Long bone deformity correction	Applied on 04.09.72 Published on 05.07.1975	March 1969 [19]
Long bone thickening	Applied on 26.03.74 Published on 25.09.1975	November 1979 [10]
Ankle joint arthrodesis	n/a	November 1976 [20]
Clubfoot	Applied on 26.03.74 Published on 25.09.1975	May 1983 [21]
Foot deformity correction and lengthening	Applied on 12.04.76 Published on 25.09.1975	November 1983 [22]
Hip disorders	Applied on 25.12.72 Published on 25.06.1978	1982 [23]
Comminuted fractures	Applied on 03.10.73 Published on 05.05.1978	January 1983 [24]

Thanks to the personality of Ilizarov and his strong character, the method overcame the prejudices and became a vivid system that advanced over time. The experimental findings of the scientific school he had created as well as practical techniques were summarized in his famous book that is still the main textbook for those orthopaedic surgeons who start training in the methods of bone lengthening and reconstruction [1]. His concepts served as guidelines for further development of the techniques and devices that have been used nowadays along with the classical Ilizarov method and apparatus [7].

Since the middle of the 1980s, the Ilizarov method has been advancing both technically and conceptually and has spread worldwide. The outcomes of its application in the 1990s and the first decade of the twenty-first century were presented by numerous studies that show the divulgation of the Ilizarov techniques across the globe [25–36]. The reports demonstrated large series of patients and concluded on the value of the Ilizarov techniques, its main disadvantages and complications. Despite good results in the majority of those studies, the authors stressed that the Ilizarov method requires adequate training to master its proper application and reduce the rate of complications [25]. Wire tract infection and a long period with the apparatus on were referred to the main drawbacks of the method. Postoperative monitoring is the key concept and means in the Ilizarov method philosophy that implies a radiographic control of bone fragment position and regeneration quality, adjustments of the frame, soft-tissue care and maintenance of joint motion [7].

## Ilizarov method in the contemporary orthopaedic practice

A search of the most recent literature in the NLM PubMed database aids to distinguish the main topics in using the Ilizarov method, and its modifications discussed by the orthopaedic community in all the orthopaedic fields where the Ilizarov method is most applicable.

### Complications due to bone injuries or consequences of their management

The Ilizarov’s ideas of external fixation appeared when he had to treat bone non-union or delayed unions in the veterans of World War II. Bone non-union and defects remains the main field where the Ilizarov method has gained undisputable honour [5–7]. He is the author of the bone fragment transport technique that was first described in 1969 [14, 15]. Nowadays, it has become a vital method for compensation of bone defects greater than 4 cm [37]. Multifocal bone transport for extensive long bone defects was studied experimentally by Ilizarov’s disciples, and the techniques of its use were presented for international readers [11, 38].

### Defects following bone tumour resection

Bone transport has been lately explored as an option of reconstruction after resection of benign and even malignant bone tumours [39]. This technique is very much relevant in the tibia where the Ilizarov fixator is surgeon and patient friendly [40].

### Bone infection

The Ilizarov method found solutions for one of the most difficult orthopaedic complications—osteomyelitis of any location in posttraumatic and postsurgical cases. Radical debridement, a special protocol of antibacterial therapy, and the antibactericidal effect that develops in tissues due to tension stress in the apparatus [41] are the conditions that enable to fight infection successfully and to reconstruct the affected bone with compression–distraction techniques. Current evidence suggests that the Ilizarov method has established itself as a gold standard for long bone infected non-union and defects [42–44]. A systemic analysis of 24 studies published on the management of infected non-union of the tibia and femur with the Ilizarov method found that the average rate of bone union was 97.26 % and infectious recurrence was 5 % [44]. Periprosthetic infection in total hip replacement that results in extensive removal of necrotic tissue has prompted the search for solution to salvage limbs. The Ilizarov apparatus could be used for this challenging situation. The modified technique of resection arthroplasty was developed at the Centre and showed promising results both in fighting infection and limb salvage [45].

### Bone lengthening and deformity correction

Despite that bone lengthening attempts had been made before the era of Ilizarov [5], bone lengthening tactics and the phenomena that undergo during this procedure were another great achievement of Prof. Ilizarov and his school researchers. The basic idea of lengthening is reproduction of the natural growth provided by the conditions of distraction under the tension stress effect that induce bone cell differentiation, expansion and morphogenesis [46]. A special automated device was invented and patented (23.09.81) that is able to produce high-frequency distraction to bring bone lengthening closer to the natural bone growth. According to research at the Centre, the regenerate formation was superior if the distraction rate of 1 mm/day was divided into smaller more frequent lengthening steps (60 daily steps of 0.017 mm each) [7, 34]. The idea resulted in several generations of automated distractors, and the use of automated lengthening was successfully reported [7, 34]. Automatic high-frequency lengthening with the Ilizarov method provided optimal conditions for faster bone tissue regeneration and a shorter treatment period. Unfortunately, automated external devices have been used in a limited number of cases due to their high costs and possible mechanical failures [47].

Ilizarov rejected intramedullary interference as he relied on the osteogenic potential of bone marrow [1]. However, the contemporary development of the lengthening methods has been subjected to the objective realities such as expenditures of the hospitals and incompliance of patients to wear an external fixator for a long time. A number of motorized intramedullary fully implantable systems have been used that follow the Ilizarov principles of distraction [48]. Such devices reduce or prevent muscle fixation and, therefore, may ease rehabilitation and increase patient comfort.

Another option is the combination of intramedullary nails or flexible hydroxyapatite (HA) coated wires with an external fixator that has been used for regenerated bone reinforcement, reduction of complication rate and duration of hospitalization [49–51]. The surgeons of the Centre introduced HA-coated intramedullary wires instead of a nail for these purposes [51]. A couple of such wires (diameter from 1.5 to 2.0 mm) is introduced from the medial and lateral sides at the metaphyseal long bone level and then pushed to the opposite metaphysis in such a way that the ray of their opposite curvature is about 40°–50°. The wires do not compromise the bone marrow content and can be easily taken out. It was proven both experimentally and clinically that such wires stimulate new endosteal bone formation and provide mechanical reinforcement and a faster period of treatment [34, 51].

During the last 10 years of the twentieth century, a revolution occurred in the management of bone deformities [35]. Ilizarov introduced the techniques of gradual deformity correction via postoperative adjustability of the external fixation. Deformity correction reinforced with flexible intramedullary HA-coated wires allows for considerable reduction of external fixation duration, decrease in the number of complications, and elimination of recurrent deformities in X-linked hereditary hypophosphatemic rickets [52]. The understanding of bone and soft-tissue regeneration has lead to a number of devices and techniques for managing simple or complex deformities, among which the Taylor spatial frame, being a computerized system, has gained a wide use [7, 34, 53].

### Rare orthopaedic conditions

The Ilizarov method showed higher union rates in treating congenital pseudarthrosis of the tibia [28, 54]. Though the united bone is of an inferior biological and mechanical quality and the refracture rates are high, the method provides a complex approach to deformity correction, lengthening and consolidation in more than a half of patients and can be considered a salvage procedure for this severe condition [54–56]. Reconstructive surgeries including centralization of the knee–ankle joint and lengthening with the Ilizarov principles have been used for such rare disorders as tibial or fibular hemimelia with satisfactory results to salvage the limb [57, 58].

### Joint arthrodesis

Despite its difficulties and the need for specific training, the Ilizarov techniques of arthrodesis provide a reliable way of achieving solid fusion with the desired angle. Advantages also include infection control, early mobilization, accurate application and possible conversion to joint replacement in case of hip arthrodesis in young patients [59–61]. Knee joint arthrodesis has shown to be applicable in infected cases after arthroplasty to salvage the limb [61].

As for ankle arthrodesis, there are situations in which a circular external fixator offers significant advantages over screw fixation. The Ilizarov ring system is indicated in difficult cases, especially when additional distal tibial pathologic conditions, bone defects, length discrepancies or the need for early weight bearing are present.

### Developmental hip disorders

Hip reconstruction using Ilizarov’s concepts is considered technically demanding and involving a lengthy period of wearing the frame. However, it was also found to be a valuable procedure for numerous neglected hip problems particularly in young patients [62, 63]. By performing the Ilizarov pelvic support osteotomy, the hip could be reserved, the limb length recovered and the gait improved significantly.

### Foot and hand pathology

Prominent contributions were made by Ilizarov to the development of techniques for foot and hand pathology management. Bloodless gradual correction of pediatric clubfoot from the age of 1 year became possible in neglected cases [27]. The Ilizarov techniques for adult multicomponent foot deformities using osteotomies offered versatility in foot position correction, enabling correction of all the components of severe deformities with three-dimensional control and lengthening of foot bones [64–66]. Ilizarov and his “hand” team invented a mini-fixator for short tubular bones that has been widely used in the Centre for management of congenital or posttraumatic disorders such as shortened hand bones, finger stumps and syndactyly [67, 68].

### Fracture repair

Among the numerous methods of long bone fracture repair, the indications to the use of the Ilizarov method are mostly high energy trauma and paraarticular fractures where open reduction and internal fixation cannot be applied [31–33, 69–72]. Temporary low profile Ilizarov apparatus application has been acknowledged as a safe procedure in cases of severe multiple injuries or polytrauma if applied by experienced surgeons or in particular cases followed by conversion into a specific assembly to address the fractures sustained but once the patient’s condition stabilizes.

## Contemporary experimental research at the Centre

A well-known drawback of the Ilizarov method such as a long wear of the apparatus resulted in the search for the ways to stimulate bone regeneration or reinforce the regenerated area. Experimental research in these directions continues and is aimed at finding better mechanical and biological solutions for faster bone formation, remodelling and reduction in treatment time.

A big animal research was dedicated to the study of intramedullary flexible HA-coated wires in bone lengthening and fracture healing which showed promising results as far as they do not compromise the osteogenic potential of bone marrow [73].

Experimental studies on the repair of fractures with different grades of bone marrow trauma showed retardation of osteoreparative processes in cases of bone cavity content removal or its severe damage [74, 75]. A method of mechanical stimulation was found applicable for long bone fracture repair in clinical settings that includes gradual distraction up to 2 mm in the early postinjury period followed by a 3-day latent period and further acute compression.

## Conclusion

The Ilizarov method has passed a long way of evolution to become an established method in the world orthopaedic practice. The impact of this method on the progress of bone lengthening and reconstruction was called revolutionary [72]. Its principles form the foundation of the contemporary bone lengthening and reconstruction surgery [5].
